# Astrocyte alterations in α-synucleinopathies

**DOI:** 10.3389/fncel.2025.1650326

**Published:** 2025-08-06

**Authors:** Manuela Rodríguez-Castañeda, Ana Campos-Ríos, Jose Antonio Lamas, Ana Covelo

**Affiliations:** ^1^Department of Functional Biology and Health Sciences, University of Vigo, Vigo, Spain; ^2^CINBIO, University of Vigo, Vigo, Spain; ^3^Laboratory of Neuroscience, Galicia Sur Health Research Institute (IIS Galicia Sur), SERGAS-UVIGO, Vigo, Spain

**Keywords:** astrocytes, tripartite synapse, α-synuclein, α-synucleinopathies, gliotransmission

## Abstract

Despite long being considered to be passive and supportive cells, in the last decades astrocytes have arisen as key regulators of neuronal excitability, synaptic transmission and plasticity. Since the discovery of the tripartite synapse, accumulating evidence suggests that astrocytes are involved in the pathogenesis of neurodegenerative diseases, including α-synucleinopathies. Here we will discuss recent evidence showing that astrocytes express endogenous α-synuclein and the implications of this protein in astrocyte cellular processes. Furthermore, we review how the expression of pathological forms of this protein in astrocytes leads to aberrant cytosolic Ca^2+^ activity in these cells and to alterations in gliotransmission and pathology progression.

## Background

Parkinson’s disease (PD), dementia with Lewy Bodies (DLB) and multiple system atrophy (MSA) are neurodegenerative diseases that belong to the class of α-synucleinopathies (Koga et al., 2021; McCann et al., 2014). These are genetic or idiopathic diseases typically characterized by disruption of motor abilities (Alafuzoff and Hartikainen, 2018), including bradykinesia, tremor and rigidity (Tolosa et al., 2021) which are commonly followed by cognitive decline in the late states of the disease (Aarsland et al., 2009; Almeida et al., 2016; Aminian and Strafella, 2013). Even though α-synucleinopathies are diagnosed based on the characteristic motor deficits, these symptoms are commonly preceded by mood disorders, such as depression, apathy, impulse control disorders and increased anxiety (Tolosa et al., 2021).

Familial genetic α-synucleinopathies are typically caused by abnormalities in the α-synuclein (α-syn) gene (*SNCA*), including duplication (Chartier-Harlin et al., 2004; Ibáñez et al., 2004), triplication (Singleton et al., 2003) or point mutations of the gene (Krüger et al., 2001; Polymeropoulos et al., 1997; Zarranz et al., 2004) that result in pathological structural modifications of the protein. These modifications lead to protein misfolding and the abnormal accumulation of α-syn in both neurons and glial cells, including astrocytes and oligodendrocytes (Ahmed et al., 2012; Brück et al., 2016; Nakamura et al., 2015). Neuronal α-syn aggregates, termed Lewy bodies (LB) and Lewy Neurites (LN), are typically found in patients with PD and DLB and constitute one of the hallmarks of these diseases (Alafuzoff and Hartikainen, 2018; Baba et al., 1998; Koga et al., 2021). On the other hand, in MSA, α-syn deposits called glial cytoplasmic inclusions (GCI) are mostly present in oligodendrocytes (Ahmed et al., 2012). Furthermore, α-syn aggregates have also been found in astrocytes at lower levels in PD, DLB and MSA (Brück et al., 2016; Wenning and Jellinger, 2005). However, whether astrocytes endogenously express α-syn or if they uptake this protein from external sources is still under debate.

Astrocytes, a type of glial cell, have arisen in the last few decades as regulators of neuronal activity, synaptic transmission, plasticity and animal behavior (Lawal et al., 2022). Although they had been classically considered passive and supportive cells, a growing body of evidence revealed that they are active elements of the tripartite synapse (Araque et al., 1999), communicating with pre- and postsynaptic terminals through gliotransmitter release and contributing to metabolic control and synapse homeostasis (Araque et al., 2014; Naveed et al., 2025; Volterra and Meldolesi, 2005). This astrocyte-neuron communication is altered under certain pathological conditions (Carter et al., 2012; Wang et al., 2023), including neurodegenerative diseases such as α-synucleinopathies (Gong et al., 2025; Ramos-Gonzalez et al., 2021). Despite these findings, most research regarding astrocyte role in the pathophysiology of these diseases has focused on their role on inflammation [reviewed in Calabresi et al. (2023)] and little is known about the contribution of gliotransmission to the pathology.

In this review, we summarize the currently available data regarding the emerging contribution of astrocytes to the pathophysiology of α-synucleinopathies. We examine current evidence demonstrating that astrocytes endogenously express α-syn in both physiological and pathological conditions. Additionally, we provide an overview of the alterations in Ca^2+^ signaling and gliotransmission observed in mouse models of α-synucleinopathies.

## α-synuclein role in physiology and pathology

**FIGURE 1:**
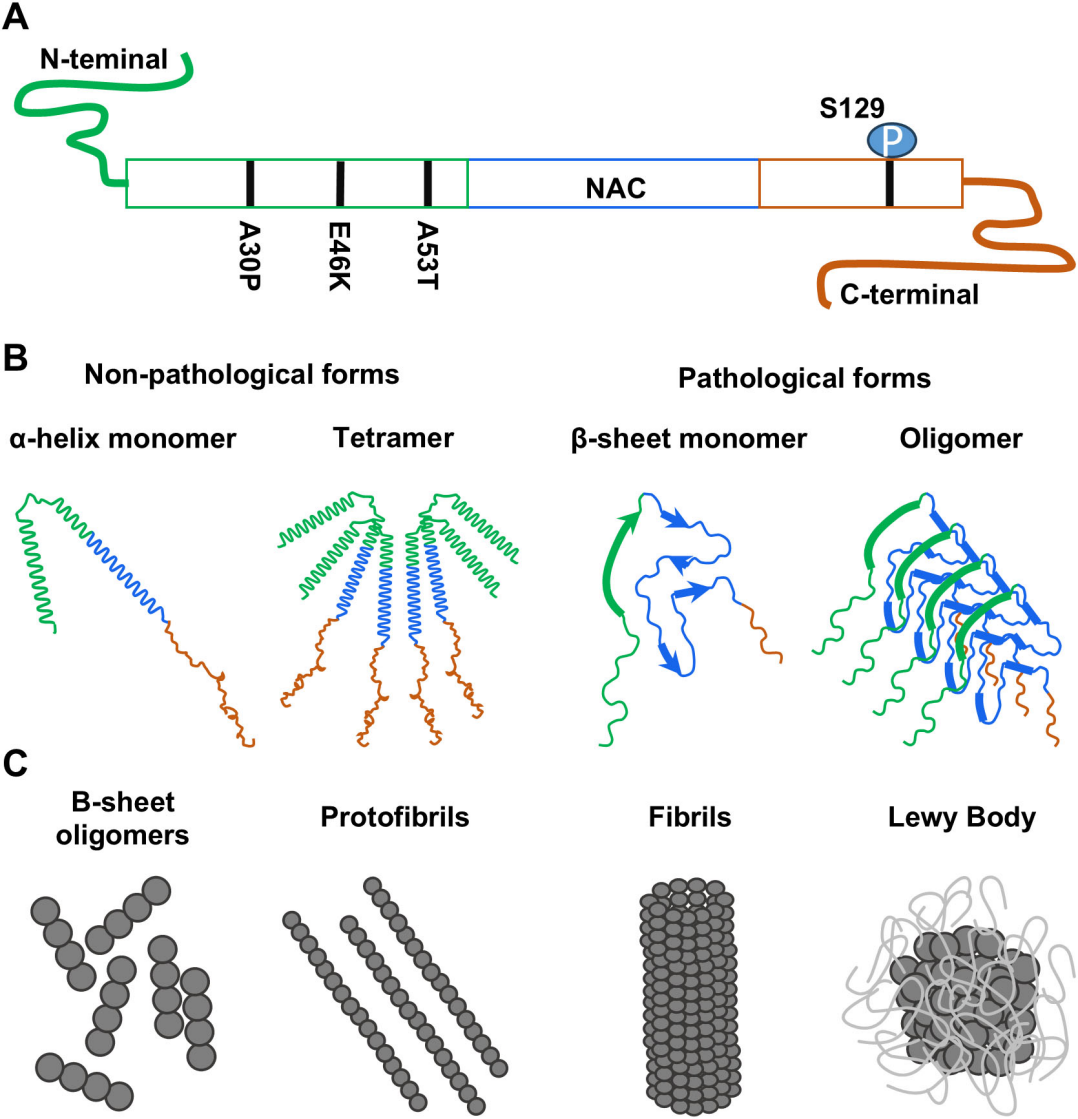
**(A)** Schematic drawing of the α-synuclein protein indicating its three domains: *N*-terminal domain (green), amyloid-binding central domain (NAC, blue) and C-terminal domain (orange). The position of the mutations and modifications mentioned in this review have been included. **(B)** Different structural conformations of α-synuclein that can be found in non-pathological and pathological conditions. **(C)** Progression in the formation of the different α-synuclein aggregates found in pathology.

α-syn was first identified in Torpedo fish electric organs and the central nervous system (CNS) of rats, primarily the hippocampus (Maroteaux and Scheller, 1991; Maroteaux et al., 1988; Schulz-Schaeffer, 2010). It is expressed along the CNS, peripheric nervous system (PNS), blood cells and other tissues (Böttner et al., 2012; Burré et al., 2018; Siebert et al., 2010). This protein is encoded by the *SNCA* gene and consists of 140 amino acids largely comprised by an 11 aminoacidic sequence repetition (Bartels et al., 2011; Maroteaux and Scheller, 1991; Maroteaux et al., 1988). α-syn presents a membrane binding *N*-terminus domain with an α-helical conformation (Vamvaca et al., 2009), a central region with aggregation propensity consisting of a non-amyloid-β component, and a C-terminus domain involved in Ca^+2^ binding and chaperone activity (Burré et al., 2018) that also regulates synaptic membrane binding (Figure 1A; Lautenschläger et al., 2018). Importantly, membrane association reduces misfolding into a β-sheet conformation. While Ca^2+^ binding to the C-terminus domain may induce *N*-terminal unfolding and aggregation-prone conformations (Tofaris, 2022). In non-pathological conditions, α-syn is enriched in neurons at presynaptic terminals (Alafuzoff and Hartikainen, 2018), where it interacts with the Soluble *N*-ethylmaleimide Attachment protein Receptor (SNARE) complex (Burré et al., 2010; Burré et al., 2014; Burré, 2015) and other synaptic proteins such as synapsin III (Zaltieri et al., 2015), and vesicular monoamine transporter 2 (VMAT2) (Guo et al., 2008). Through the interaction with these proteins, α-syn regulates neurotransmitter release by controlling vesicle trafficking and the readily releasable pools of synaptic vesicles (Cabin et al., 2002; Nemani et al., 2010). Indeed, the overexpression of α-syn leads to a decrease in neurotransmission due to a reduction in vesicle trafficking and, therefore, the recovery of the docked vesicles that form the readily releasable pools (Burré et al., 2014). Consistently, mouse models where α-syn has been knocked-out show an increase in the paired-pulse ratio (Zaltieri et al., 2015), indicative of an increase in the readily releasable pools. Additionally, α-syn knock-out mice display decrease neurotransmission under prolonged repetitive stimuli, suggesting that the increase vesicle trafficking leads to the quick depletion of both the docked and reserve pool vesicles (Burré et al., 2010). α-syn can also be found in specific organelles such as the nucleus (Maroteaux et al., 1988), mitochondria (Li et al., 2007), endoplasmic reticulum (Hoozemans et al., 2007), Golgi apparatus (Gosavi et al., 2002), and in the endolysosomal system (Lee et al., 2004). Yet, its functions in the different cellular and subcellular compartments are not fully understood.

In physiological conditions, α-syn coexists in two non-pathological forms: a small soluble unfolded monomer with α-helical structure, and an insoluble tetramer with β-sheet structure and no amyloid-like aggregation potential (Figure 1B), which is commonly associated to synaptosomes (Bartels et al., 2011; Siebert et al., 2010). In non-pathological conditions, these two structural forms of α-syn are kept in balance. However, during pathological processes, α-syn monomers can adopt a neurotoxic β-sheet conformation when it dissociates from membranes (Burré et al., 2015; Nath et al., 2010). This conformation has self-assembly capacity and promotes the generation of α-syn oligomers that can further develop into protofibrils (Mehra et al., 2022; Vilar et al., 2008), mature fibrils (Lee et al., 2004) and finally leads to the formation of LB (Figure 1C) [reviewed in Li et al. (2022)]. The distribution and maturation of this α-syn aggregations in the brain is particularly important, as it serves as indicative for the stage of the disease (Braak et al., 2007).

A mutated, pathological α-syn form was first reported forming the LB in patients with a familial form of PD (Figure 1A) (Polymeropoulos et al., 1997; Spillantini et al., 1997). This mutation corresponded to a change of a guanine to an adenosine in position 209 (G209A) in the *SNCA* gene that resulted in an alanine to threonine substitution (A53T) in the amino acid chain (Elobeid et al., 2016; Parkkinen et al., 2008; Zaccai et al., 2015) and shows increased aggregation when compared with wild-type α-syn *in vitro* (Conway et al., 1998). Later, other point mutations associated to autosomal dominant PD and LBD (such as A30P and E46K amino acid changes) were also found (Krüger et al., 1998; Zarranz et al., 2004). Additionally, modifications in the amino acid chain, such as the phosphorylation of S129 (Anderson et al., 2006), were described in α-syn aggregates of LBD patients, which although is not necessary for the LB formation, might induce a more severe pathology (Conway et al., 1998; Karampetsou et al., 2017; Parkkinen et al., 2008; Sano et al., 2021). Noteworthy, aberrations in α-syn are not restricted to the CNS as α-syn aggregations have been found at different levels of the nervous system, including the cerebrospinal fluid, as well as in peripheral organs such as the gastrointestinal track and at blood cells (Li et al., 2022).

Interestingly, experiments performed in cell cultures revealed that the addition of α-syn with the point mutation S129 can induce the aggregation of endogenous α-syn, revealing the capability of this protein of transmitting from cell to cell (Lee and Masliah, 2015). Additionally, it has been observed that exosomes from patients with DLB induce the seeding and propagation of α-syn aggregates in healthy mice and *in vitro* experiments (Ngolab et al., 2017). These data suggest that pathological forms of α-syn can spread in a prion-like manner (Chen and Mor, 2023; Espinosa-Oliva et al., 2024; Uemura et al., 2018), which seems to induce different pathological symptoms depending on the type of α-syn deposit (Chen and Mor, 2023; Espinosa-Oliva et al., 2024; Uemura et al., 2018). For instance, the conformation and biology of the α-syn deposits differ between the brains of human patients with MSA and LBD (Peng et al., 2018). Accordingly, the injection of different forms of α-syn – such as oligomers, or fibrils – into the substantia nigra has been shown to produce distinct histopathological profiles resembling with either PD or MSA, being the administration of fibrillary α-syn the one that produced a stronger pathology (Peelaerts et al., 2015).

## Astrocytes are key controllers of brain activity

In the human brain, the ratio of glial cells to neurons is of approximately 1:1, with oligodendrocytes and astrocytes being the most abundant of these cells (Bahney and Von Bartheld, 2014; Bahney and von Bartheld, 2018; Pakkenberg and Gundersen, 1988; von Bartheld et al., 2016). Astrocytes have a close and dynamic relationship with synapses, as they physically enwrap and interact with them (Ventura and Harris, 1999). This physical association allows astrocytes to perform extremely important roles such as maintaining homeostatic control of certain ions (Lia et al., 2023; Murakami and Kurachi, 2016), re-uptaking neurotransmitters, participating in neurogenesis and synaptogenesis, and providing metabolic support to neurons (Czopka et al., 2020; Dallérac et al., 2018; Naveed et al., 2025). Additionally, astrocytes express a wide range of G protein-coupled receptors (GPCRs) that can be activated by neurotransmitters and trigger intracellular signaling pathways that lead to the production of inositol (Aarsland et al., 2009; Koga et al., 2021; Tolosa et al., 2021)-triphosphate (IP_3_) and Ca^2+^ exit from internal stores to the cytosol (Kim et al., 2025; Squadrani et al., 2024; Volterra and Meldolesi, 2005). These Ca^2+^ increases in the cytosol consequently lead to the release of different neuroactive substances, so called gliotransmitters, such as glutamate, purines or D-serine. Through the release of gliotransmitters, astrocytes can communicate with other cells, including neurons, leading to the regulation of neuronal activity, synaptic transmission, plasticity and behavior (Araque et al., 2014; Liu et al., 2021; Volterra and Meldolesi, 2005). Noteworthy, the release of the different types of gliotransmitters seems to depend on the stimuli that the astrocyte receives (Covelo and Araque, 2018) which may vary depending on the brain region studied. Thus, astrocytes are now recognized as crucial players in the regulation of brain circuits, bridging neuronal activity, metabolism and homeostasis (Dallérac et al., 2018; Kim et al., 2025; Squadrani et al., 2024).

Importantly, astrocytes occupy separate anatomical domains with minimal overlap between astrocytic processes (Bushong et al., 2002; Wilhelmsson et al., 2006). Thus, it is well accepted in the field that one synapse can be contacted and regulated uniquely by a single astrocyte (Halassa et al., 2007). Furthermore, single astrocytes are in contact with hundred of thousands of synapses within their domains in rodents (Ventura and Harris, 1999), while that number can reach up to two million synapses in the human brain (Oberheim et al., 2009). This highlights the potential of these cells on the regulation of neuronal network activity and their potential impact during pathological processes.

## α-syn expression in astrocytes

Glial, and particularly astrocyte, contribution to α-synucleinopathies is starting to get attention as targets in diseases like Parkinson (Chen et al., 2025). Although first reported in neurons, α-syn aggregates have been also found in astrocytes in post-mortem brains of human patients with PD, DLB and MSA (Altay et al., 2022; Braak et al., 2007; Terada et al., 2003; Wakabayashi et al., 2000) and mouse models of α-synucleinopathies (Sacino et al., 2013; Sorrentino et al., 2017), suggesting that these cells may play a role in the pathology (Carter et al., 2012; Halassa et al., 2007; Oberheim et al., 2009; Ventura and Harris, 1999). A recent study performed in human brain tissue of PD, BLD and MSA patients has demonstrated that astroglial α-syn accumulations express structural modification when compared with neuronal ones (Altay et al., 2022), indicating that protein modification may take place in the astrocytes.

**FIGURE 2:**
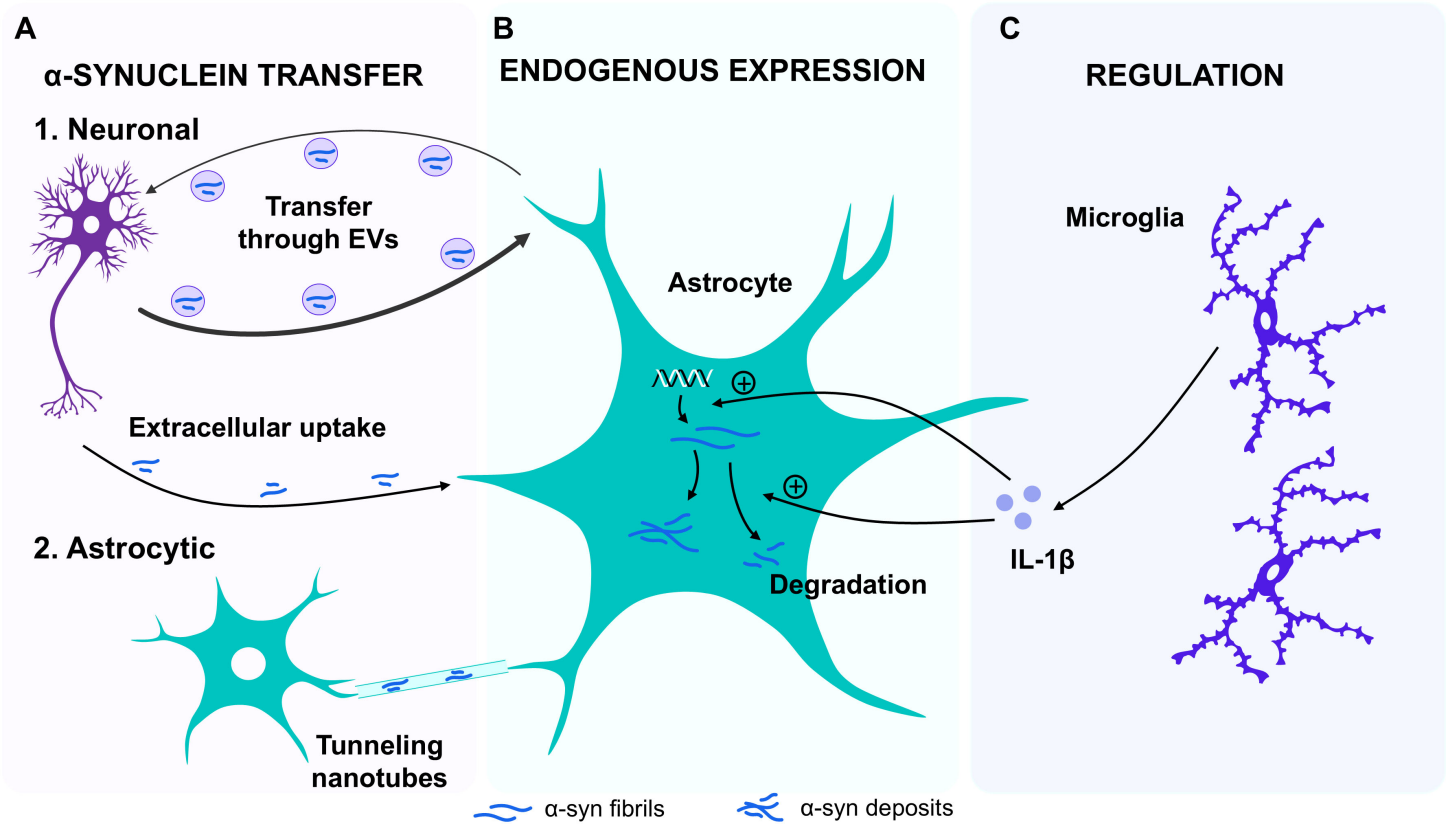
α-synuclein transfer, degradation, expression and regulation between astrocytes and other cells. **(A)** α-synuclein (α-syn) transfer. It has been established that the process of transfer occurs between neurons and astrocytes via the uptake of aggregates of α-syn from the extracellular medium through endosomes. This transfer is bidirectional, yet it is less efficient in the astrocyte-to-neuron direction. Furthermore, direct transfer from the medium also appears to be possible, as well as a recently discovered mechanism whereby transfer between astrocytes can occur through tunneling nanotubes. **(B)** Endogenous expression: The expression of α-syn protein has been observed in cultured mouse astrocytes and in a human astrocyte cell line. The expression of both α-syn mRNA and protein has been observed to increase upon exposure to interleukin 1β (IL-1β) in culture. In addition, it has been demonstrated that astrocytes are capable of degrading α-syn aggregates, a capacity that is augmented following IL-1β exposure. **(C)** Regulation: Recent studies suggest that the surrounding cellular environment may also influence this ability to degrade α-syn aggregates. When astrocyte cultures are co-cultured with microglia, the degradation of α-syn aggregates increases, likely due to the release of interleukins such as IL-1β.

The origin of the α-syn inclusions found in astrocytes have long been under debate. One of the hypotheses is that astrocytes can uptake α-syn produced by neurons (Figure 2A). In line with this, α-syn inclusions were found in astrocytes in a mouse model that selectively express a mutated form of α-syn (human A30P mutation) exclusively in neurons (Marxreiter et al., 2013) supporting the idea that α-syn can be transferred from neurons to astrocytes. Indeed, it has been observed that α-syn released from neurons can be taken up by astrocytes through endocytosis, leading to an astroglial inflammatory response, alterations in astrocyte gene expression and contributing to astroglial pathology (Lee et al., 2010b). Additionally, a direct transmission of α-syn aggregates from neurons to astrocytes via extracellular vesicles (EVs) has been reported (Lee et al., 2010b; Meng et al., 2020). Importantly, the neuron-to-astrocyte α-syn transfer may be important for the clearance of α-syn deposits, since astrocytes seem capable of degrading α-syn fibrils, a property not observed in neurons (Loria et al., 2017). The capacity of astrocytes of reducing α-syn deposits in cultures is increased in the presence of microglia (Rostami et al., 2020; Rostami et al., 2021), suggesting that environmental factors are key regulators of this process. Additionally, in mouse models of α-synucleinopathies, the deletion of the circadian clock protein BMAL1 in astrocytes showed increased α-syn phagocytosis and decreased spreading of the pathology (Sheehan et al., 2023), while astrocytes carrying the G2019S mutation exhibited lower capacity to internalize and degrade α-syn fibrils via endo-lysosomal pathways (Anderson et al., 2006). These studies demonstrate that astrocytes play a key role in the uptake and degradation of α-syn aggregates, a phenomenon that appears to be region-dependent (Basurco et al., 2023). This is probably due to the astrocytic heterogeneity that exists between different brain regions, which seems to be adapted to the diversity of neuronal types and the functional requirements [reviewed in Haim et al. (2017)]. Regarding the midbrain, where loss of dopaminergic neurons occurs during PD, it has been observed that a specific molecular and functional profile of astrocytes influences dopaminergic neurons activity, and that the transplantation of healthy cultured astrocytes from this brain region into a PD mouse model facilitated the clearance of toxic α-syn aggregates (Yang et al., 2022). This highlights astrocyte potential role in managing protein accumulation and demonstrates the importance of glial cells in the progression of α-synucleinopathies. Noteworthy, the intercellular transfer of α-syn is not restricted only to a neuron-to-astrocyte transfer as an efficient astrocyte-to-astrocyte transfer of α-syn through tunneling nanotubes has been demonstrated *in vitro* and *in vivo* (Rostami et al., 2017). Additionally, astrocyte-to-neuron transfer of α-syn has also been observed *ex vivo* and in cell cultures, although it seems to be less efficient (Loria et al., 2017). Interestingly, single-cell RNA sequencing from astrocytes derived from A53T-mutant mice revealed that the internalization of α-syn aggregates induced the secretion of proinflammatory cytokines (TNF-α and interleukin 6) from astrocytes, generating a reactive environment and activating a reactive crosstalk communication with neurons that lead to neurodegeneration (Li et al., 2024). Furthermore, single-cell transcriptomics in PD human post-mortem substantia nigra samples showed a specific astrocyte subpopulation enriched in proinflammatory signaling and elevated cytokine activity (Gong et al., 2025), a reactive phenotype triggered by α-syn internalization (Lee et al., 2010b).

In addition to the α-syn that is up taken from the extracellular media or transferred from other cells, increasing evidence suggests that astrocytes can also express this protein in an autonomous manner (Figure 2B). Accordingly, the expression of α-syn has been detected in pure astrocyte primary cultures obtained from mice (Gu et al., 2010; Nanclares et al., 2023) and it has been shown that cell lines of human cultured astrocytes expressed both α-syn mRNA and protein (Tanji et al., 2001). Interestingly, this expression is increased by astrocyte exposure to interleukin 1 β (IL-1β) (Tanji et al., 2001), a cytokine closely related to inflammation and astrocyte proliferation that is increased in dopaminergic and striatal regions of human PD brains (Jafariaghdam et al., 2024; Saghazadeh et al., 2016) suggesting that α-syn expression in astrocytes is influenced by external stimuli (Figure 2C).

## Role of α-syn in astrocyte physiology

The role of α-syn in astrocytes under normal conditions is still unclear. As described above, neuronal α-syn is involved in vesicle release by interacting with the SNARE complex and other synaptic proteins involved in vesicle release (Burré et al., 2010; Burré et al., 2014; Burré, 2015; Guo et al., 2008; Zaltieri et al., 2015). Thus, it is possible that astroglial α-syn may interact with these proteins in astrocytes as well. Indeed, a mouse line that overexpresses A53T-mutant α-syn displays increased glutamate release from astrocytes (Nanclares et al., 2023), suggesting that α-syn may be involved in the regulation of gliotransmitter release. However, the molecular mechanisms underlying these alterations in gliotransmission remains to be elucidated.

Experiments performed in α-syn gene-ablated mice found disrupted fatty acid uptake and trafficking in astrocytes, indicating that α-syn may be involved in the regulation of fatty acid metabolism in these cells (Castagnet et al., 2005). Indeed, cultured human astrocytes derived from induced pluripotent stem cells obtained from human patients of PD showed increased expression of α-syn and altered metabolism (Sonninen et al., 2020). This role in metabolism is further supported by the fact that astrocyte exposure to α-syn oligomers leads to mitochondrial dysfunction, resulting in increased oxygen consumption, altered iron status and cell death (Braidy et al., 2013; Cui et al., 2020). Another study revealed that astrocytes carrying the G2019S mutation exhibited alterations in mitochondrial morphology and activity, accompanied by elevated oxidative stress and decreasing their participation in the homeostasis control in comparison to those observed in healthy patients (Ramos-Gonzalez et al., 2021). In addition, the overexpression of A53T-mutant α-syn in astrocytes triggered endoplasmic reticulum stress, Golgi apparatus fragmentation and apoptosis due to the excess and misfolding of the protein (Liu et al., 2018) suggesting that the proper handling of α-syn by astrocytes is key for cell survival. Research on primary astrocytes exposed to α-syn aggregates revealed an increase in the secretion of extracellular vesicles (EVs), which could be a response to α-syn-induced lysosomal dysfunction (Wang et al., 2023). Furthermore, peripheral erythrocyte-derived EVs from PD patients carrying α-syn can trespass the blood brain barrier and accumulate in astrocyte endfeet, impairing astrocytic glutamate uptake and leading to synaptic dysfunction (Sheng et al., 2020). Importantly, patients with PD showed significantly higher levels of astrocytes EVs than those from healthy control group, suggesting that this could be a potential biomarker for diagnosis or differential diagnosis of this disease (Wang et al., 2023). On the other hand, a recent study showed that healthy midbrain astrocytes secreted EVs that rescued neuronal death and preserved mitochondrial function in a PD model (Leggio et al., 2022), implying a protective role of astrocytic EVs. Furthermore, EVs derived from fibroblast growth factor 2, key for astrocytic development, have been also shown to reserve the reactive phenotype in astrocytes and to enhance synaptogenesis in PD mice models (Wen et al., 2025), being key contributors for neuroprotection. Thus, even though astrocyte-derived EVs seems to play an important role in the development of the disease, their exact function remains unclear. Current evidence suggest that they may exert both neurotoxic and neuroprotective roles, possibly depending on the context, disease stage or molecular content, highlighting the need of further research in this topic.

Additionally, accumulating evidence suggests that α-syn may play a role in astrocyte reactivity which, in turn, may contribute to the neuroinflammation and neuronal death found in α-synucleinopathies (Chou et al., 2021; Tanji et al., 2001). Recent studies have found that exposure of astrocytes to α-syn leads to neurotoxic activation of astrocytes and neuroinflammation through the release of interleukins and chemokines (Chou et al., 2021; Lee et al., 2010a). The expression of A53T-mutant α-syn specifically in astrocytes lead to severe astrogliosis showing increased GFAP expression, morphological modifications and the release of pro-inflammatory factors (Gu et al., 2010). Additionally, the inhibition of astrocyte reactivity decreased neuronal loss and increased the life expectancy in a mouse line that expressed this same mutation (Yun et al., 2018). On the other hand, astrocytes are also capable of contributing to neuroprotection when exposed to exogenous α-syn. In a mouse model of PD, the activation of cannabinoid type 2 receptors (CB2R) on astrocytes induces the degradation of inflammasomes through autophagy (Zhu et al., 2023) and, therefore, to a reduction in the release of interleukins (Mangan et al., 2018). Additionally, the release of anti-inflammatory factors has also been observed, although this seems to occur at later stages (Lee et al., 2010a). Therefore, although it seems that astrocytes play a key role in the progression of the disease by regulating neuroinflammation, it has been proposed that their involvement may be different depending on the spatiotemporal properties of α-syn pathology (Ozoran and Srinivasan, 2023).

Importantly, α-syn aggregates and iron accumulations have been found in astrocytes of the spinal cord in humans that had undergone traumatic spinal cord injury (SCI), as well as in a mouse model of SCI (Sauerbeck et al., 2021). Interestingly, under SCI the knock-out of the *SNCA* gene resulted in neuroprotection and decreased pro-inflammatory factors (Sauerbeck et al., 2021), suggesting that indeed α-syn may be playing a role in the induction of astrocyte reactivity and neuroinflammation in response to insults not even related to α-synucleinopathies. Similarly, astrocyte-derived pro-inflammatory signals lead to α-syn aggregation in the brain of mice undergoing a viral encephalitis (Bantle et al., 2021). These findings reveal the complex interactions between astrocytes and α-syn in the progression of neurodegenerative diseases, emphasizing the role of astrocytes in mediating inflammatory responses.

## α-syn effects in astrocyte Ca^2+^ signaling and in gliotransmission

**FIGURE 3:**
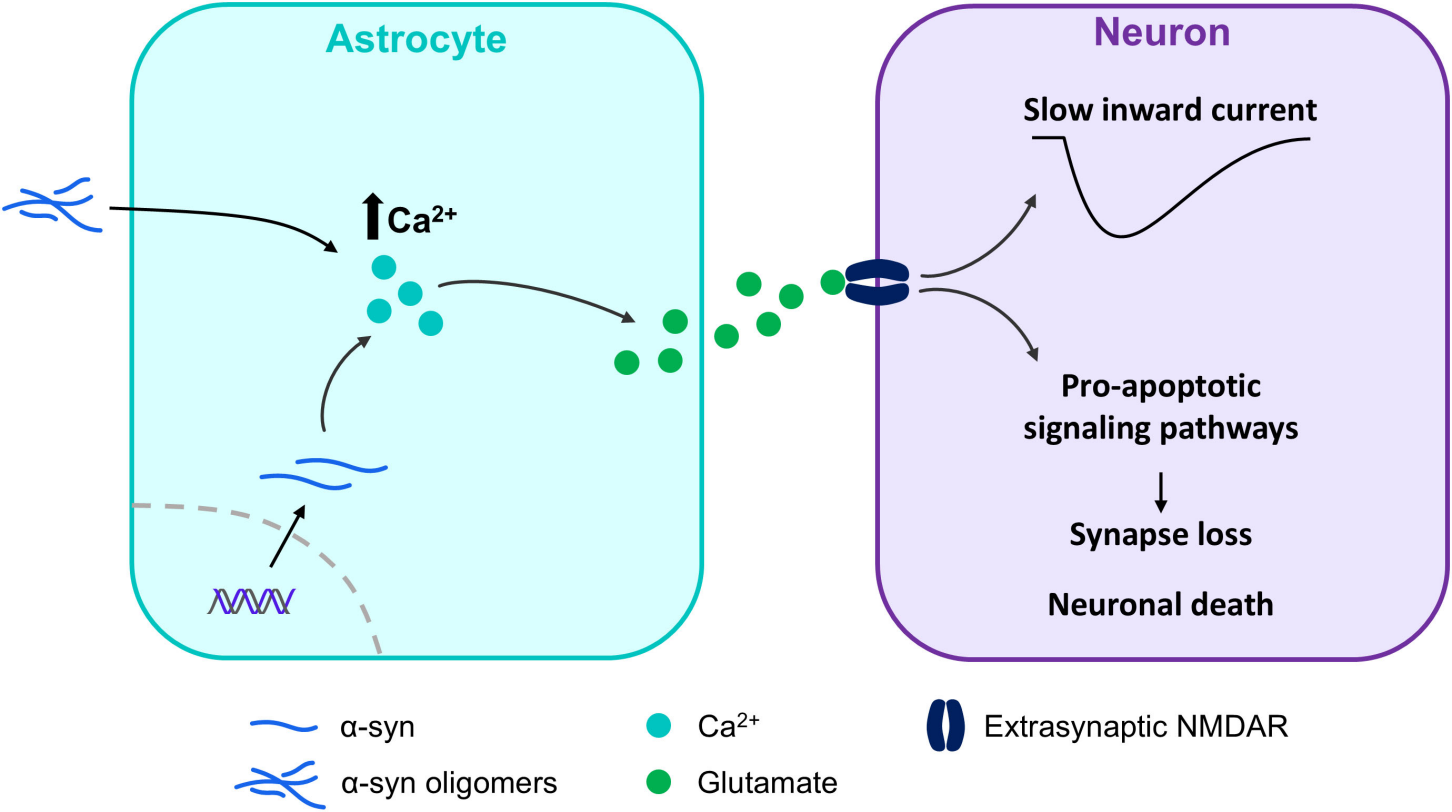
α-synuclein and their potential role in the regulation of cytosolic Ca^2+^. α-synuclein (α-syn) oligomers and overexpression of α-syn induce Ca^2+^ dependent glutamate release in astrocytes, leading to extrasynaptic N-methyl-D-aspartate (NMDA) receptors activation in neurons. This triggers the activation of pro-apoptotic signaling pathways, ultimately resulting in synaptic loss and neuronal death. Furthermore, the activation of these receptors mediates slow inward currents (SICs) in neurons, which can be used to assay astrocyte-derived glutamate release.

Cytosolic astrocyte Ca^2+^ activity is essential for proper brain function as it is involved in numerous aspects of astrocyte physiology, including the integration of neuronal and metabolic signals, cellular communication, neurovascular coupling and gliotransmitter release(Goenaga et al., 2023; Novakovic and Prakriya, 2025; Veiga et al., 2025). Those Ca^2+^ signals are altered under different pathologies (Baraibar et al., 2024; Khakh et al., 2017; Nanclares et al., 2021; Rakers and Petzold, 2017), suggesting that astrocytes contribute to disease not only through neuroinflammation, but also through alterations in astrocyte-to-neuron communication. Regarding, α-synucleinopathies, experiments performed in hippocampal acute slices from transgenic mouse models of familial PD expressing A53T-mutant α-syn showed that cytosolic astrocyte Ca^+2^ activity was increased when compared with non-transgenic mice (Nanclares et al., 2023). This increase in Ca^+2^ activity was independent of neurotransmitter receptor activation and not due to changes in neuronal signaling, indicating that this dysregulation in astrocyte Ca^+2^ activity is cell autonomous (Nanclares et al., 2023). Interestingly, these effects were observed in acute slices obtained from mice overexpressing A53T-mutant α-syn, but not from mice overexpressing similar levels of wild type α-syn or α-syn with other point mutations, such as A30P. Importantly, these effects are due to the endogenous expression of α-syn in astrocytes, as astrocyte Ca^2+^ signaling was normal in a mouse model overexpressing A53T-mutant α-syn exclusively in neurons (Nanclares et al., 2023). Additionally, astrocytes derived from induced pluripotent stem cells of patients with PD presented increased expression of α-syn mRNA and protein, which resulted in increased Ca^2+^ activity and inflammatory responses with higher cytokine release (Sonninen et al., 2020). Furthermore, cultured human astrocytes show increased Ca^2+^ activity when exposed to α-syn oligomers (Trudler et al., 2021). Interestingly, the exposure of astrocyte and microglial co-cultures to α-syn oligomers triggers persistent nuclear factor ϰB (NF-ϰB) signaling onto astrocytes that lead to an increase in the expression of Ca^2+^ channels (Leandrou et al., 2024). Thus, even though the role of α-syn in astrocytes is not fully understood, it seems to play a central role in the regulation of cytosolic Ca^2+^ (Figure 3).

Glutamate release from astrocytes was also augmented in the hippocampus of mice overexpressing A53T-mutant α-syn, generating depolarizing slow inward currents (SICs) in neurons (Nanclares et al., 2023) and indicating that α-syn is also involved in the release of gliotransmitters. This astrocytic glutamate specifically signaled into extrasynaptic N-metyl-D-Aspartate receptors (NMDA) (Fellin et al., 2004; Nanclares et al., 2023), whose activation is associated with apoptotic signaling cascades in certain pathologies (Chou et al., 2021), including PD (Ahmed et al., 2011; Veiga et al., 2025; Wang et al., 2020). Consistently, exposure of cultured mouse astrocytes to α-syn oligomers, induces a tonic release of glutamate that specifically signals into extrasynaptic NMDA receptors and is associated with synapse loss and the expansion of the disease (Trudler et al., 2021). Interestingly, protein levels of mitogen-activated protein kinase (MAPK) p38 are increased in astrocytes in both mice overexpressing α-syn and in patients with DLB (Iba et al., 2020). This is particularly important, as p38 has been suggested to underlie glutamate release from astrocytes in the hippocampus, playing a crucial role in the induction of synaptic plasticity (Navarrete et al., 2019). In addition to the increased glutamate release from astrocytes, experiments performed in a double transgenic mouse line selectively expressing A53T in astrocytes showed a decreased expression of glutamate transporters (Gu et al., 2010), possibly leading to reduced glutamate clearance from the synapse and resulting in further excitotoxicity. Interestingly, these mice showed dopaminergic and motor neuron loss in the brainstem and midbrain that was associated to motor disco-ordination and movement disabilities (Gu et al., 2010), stressing the importance of astrocyte involvement in the pathogenesis of α-synucleinopathies.

## Conclusion

Astrocytes carry out a variety of functions in the brain, ranging from providing metabolic support to neurons to the regulation of neuronal activity. While astrocyte-to-neuron communication is key for normal brain function, this topic has received little attention under pathological conditions, including α-synucleinopathies. Indeed, we have recently started to understand that astrocytes directly contribute to the pathological progression of these diseases by regulating α-syn uptake from the extracellular space, the degradation of α-syn aggregates and the generation of the immune response. However, the role of astrocyte Ca^2+^ signal and gliotransmission in α-synucleinopathies is still far from being elucidated. Here, we discussed some of the few studies that have addressed this topic. These studies have revealed that pathological forms of α-syn alter astrocyte Ca^2+^ signals leading to aberrant glutamate release and neurotoxicity, stressing astrocytes as potential targets for therapeutic strategies for α-synucleinopathies treatment.
